# Fluid flow facilitates inward rectifier K^+^ current by convectively restoring [K^+^] at the cell membrane surface

**DOI:** 10.1038/srep39585

**Published:** 2016-12-22

**Authors:** Jae Gon Kim, Sang Woong Park, Doyoung Byun, Wahn Soo Choi, Dong Jun Sung, Kyung Chul Shin, Hyun-ji Kim, Young-Eun Leem, Jong-Sun Kang, Hana Cho, Bokyung Kim, Sung I Cho, Young Min Bae

**Affiliations:** 1 Department of Physiology, KU Open Innovation Center, Research Institute of Medical Science, Konkuk University School of Medicine, Chungju, Chungbuk 380-701, South Korea; 2 Department of Emergency Medical Services, Eulji University, Seongnam, Gyeonggi-do, 461-713, South Korea; 3 Department of Mechanical Engineering, Sungkyunkwan University, 2066 Seobu-Ro, Jangan-Gu, Suwon, Gyeonggi, 440-746, South Korea; 4 Department of Immunology, School of Medicine, Konkuk University, Chungju 380-701, South Korea; 5 Division of Sport Science, College of Science and Technology, Konkuk University, Chungju, 380-701, South Korea; 6 Department of Physiology and Samsung Biomedical Research Institute, Sungkyunkwan University School of Medicine, Suwon, 440-746, South Korea; 7 Department of Molecular Cell Biology and Samsung Biomedical Research Institute, Sungkyunkwan University School of Medicine, Suwon, 440-746, South Korea

## Abstract

The inward rectifier Kir2.1 current (IKir2.1) was reported to be facilitated by fluid flow. However, the mechanism underlying this facilitation remains uncertain. We hypothesized that during K^+^ influx or efflux, [K^+^] adjacent to the outer mouth of the Kir2.1 channel might decrease or increase, respectively, compared with the average [K^+^] of the bulk extracellular solution, and that fluid flow could restore the original [K^+^] and result in the apparent facilitation of IKir2.1. We recorded the IKir2.1 in RBL-2H3 cells and HEK293T cells that were ectopically over-expressed with Kir2.1 channels by using the whole-cell patch-clamp technique. Fluid-flow application immediately increased the IKir2.1, which was not prevented by either the pretreatment with inhibitors of various protein kinases or the modulation of the cytoskeleton and caveolae. The magnitudes of the increases of IKir2.1 by fluid flow were driving force-dependent. Simulations performed using the Nernst-Planck mass equation indicated that [K^+^] near the membrane surface fell markedly below the average [K^+^] of the bulk extracellular solution during K^+^ influx, and, notably, that fluid flow restored the decreased [K^+^] at the cell surface in a flow rate-dependent manner. These results support the “convection-regulation hypothesis” and define a novel interpretation of fluid flow-induced modulation of ion channels.

Fluid flow is a critical mechanical stimulus in living systems that generates mechanical shear forces and regulates the activities of numerous crucial proteins. The fluid flow-induced shear force has been reported to regulate ion channels, cytoskeleton networks, and signaling molecules such as G proteins, tyrosine kinases, mitogen-activated protein kinases, and extracellular signal-regulated kinases^1^,^2^,^3^,^4^,^5^. Specifically, in endothelial cells, fluid flow (or shear stress) was reported to regulate vascular tone and vascular homeostasis by activating endothelial nitric oxide (NO) synthase and ion channels^6^,^7^. In ventricular cardiomyocytes, fluid flow decreased the L-type Ca^2+^ current by increasing Ca^2+^ release from the sarcoplasmic reticulum^8^, whereas in vascular myocytes, the L-type Ca^2+^ current was facilitated by fluid flow^9^,^10^. In mast cells, degranulation and histamine release were mediated by Ca^2+^ influx through vanilloid receptor transient receptor potential-4 channels, which were reported to be activated by shear stress^11^.

Inward rectifier Kir2.1 channel functions as a typical Kir channel, and it is expressed in diverse types of cells such as ventricular cardiomyocytes, vascular endothelial cells, neurons, and blood cells such as mast cells. In ventricular myocytes, Kir2.1 largely contributes to maintaining the resting membrane potential (E_m_). In endothelial cells, the concomitant activation of Kir channels and Ca^2+^ -activated K^+^ channels during agonist- or mechanical stimulus-induced endothelial cell activation contributes toward providing the driving force for Ca^2+^. Blockade of endothelial Kir channels by barium chloride inhibited both flow-induced Ca^2+^ influx and Ca^2+^ -dependent production of NO^12^,^13^.

Kir2.1 contains potential serine/threonine and tyrosine phosphorylation sites and was reported to be regulated by PKA, PKC, and PTK^14^,^15^,^16^,^17^. Hoger *et al*.^18^ reported that endothelial Kir2.1, which was heterologously expressed in oocytes or human embryonic kidney (HEK)-293 cells, was activated by fluid flow-induced shear force; Hoger and coworkers suggested that tyrosine phosphorylation of Kir2.1 (at Y242 and Y366) mediated the shear force-induced activation of Kir2.1. However, the mechanism of flow-induced Kir2.1 activation remains incompletely elucidated.

The movement of ions in solution, where an electrochemical gradient is present, can occur through diffusion (movement induced by the concentration gradient), migration (movement driven by the electrical gradient), and convection (movement through convection or fluid flow). Park *et al*.^10^ previously reported that the convection mode was >1,000 times more dominant than diffusion or migration. Because of this fluid flow-induced convective transport of Cl^−^ ions, during patch-clamp recordings, the junction potential between the bath solution and the Ag/AgCl ground electrode was distinct under static and fluid-flow conditions^10^. This phenomenon might also be observed between Kir2.1 channels and the bath solution (extracellular fluids such as the interstitial fluid or blood plasma in a clinical setting) during K^+^ current generation, because the Kir2.1 channel can function as a K^+^ electrode in the same manner as the Ag/AgCl electrode functions as a Cl^−^ electrode. Provided that the Kir2.1 conductance is “sufficiently large” and the open time is “adequately long” and thus the K^+^ influx is “sufficiently rapid and sustained,” [K^+^] at the channel inlet (outer mouth) might fall below the average [K^+^] of the bulk extracellular solution. Here, it is possible that the electrochemical gradient-dependent diffusion of K^+^ from the bulk solution to this “microdomain” of the channel inlet (outer mouth) alone is not sufficient. In this case, fluid flow could provide convective transport of K^+^ from the bulk solution to the “microdomain” of the channel inlet in the same manner as fluid flow did at the Ag/AgCl electrode surface^10^. The Kir2.1 channel exhibits a large single-channel conductance (25−36 pS) and a long open time (time constant from the dwell-time histogram of open channels: ~240 ms at −100 mV)^19^,^20^,^21^. Based on these findings, we hypothesized that [K^+^] adjacent to the channel inlet of the Kir2.1 channel conduction pore might fall below the average [K^+^] of the bulk extracellular solution.

Here, we provide evidence indicating that fluid flow increases the Kir2.1 current (IKir2.1) independently of cellular signaling molecules such as protein kinases, the cytoskeleton structure, and caveolar integrity. Moreover, the Nernst-Planck mass-flux equation predicted a steep concentration gradient between the membrane surface of an artificial cell, whose electrophysiological properties were similar to those of RBL-2H3 cells, and the bulk extracellular solution during K^+^ influx, and the simulation also predicted a drastic recovery of this concentration gradient as a result of fluid flow. We suggest that a [K^+^] gradient is generated between the bulk extracellular solution and the membrane surface of the channel inlet during K^+^ influx, and that fluid flow increases the IKir2.1 through convective restoration of the original [K^+^] adjacent to the channel inlet independently of cellular signaling and channel gating modulation. These findings support a novel interpretation of fluid flow-induced modulation of ion channels.

## Results

### Fluid flow increases the IKir2.1 in RBL-2H3 cells

**Figure 1:**
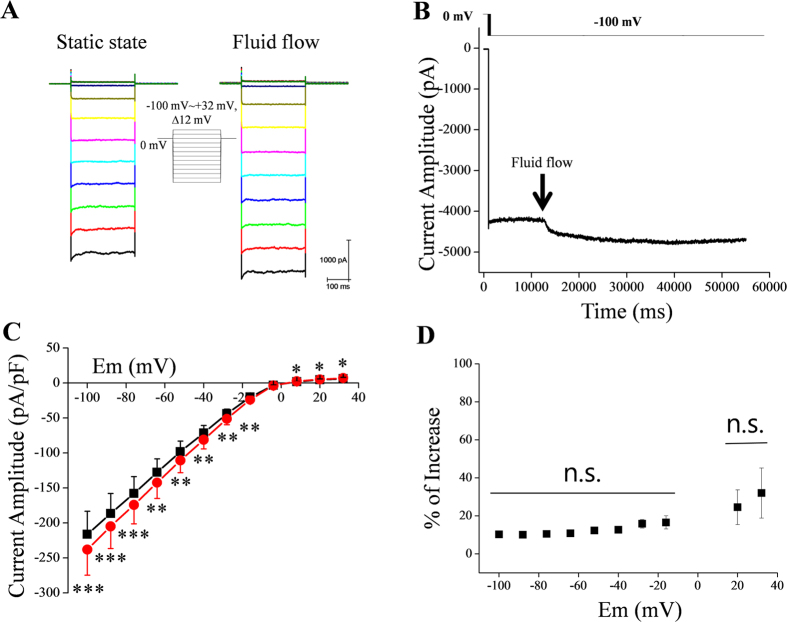
IKir2.1 in RBL-2H3 cells is increased by fluid flow. (**A**) Representative Kir2.1 currents elicited by voltage steps starting from a holding potential of 0 mV, under control (static) and fluid-flow conditions in a high-extracellular-K^+^ solution. (**B**) Representative tracing of IKir2.1 at −100 mV before and after fluid-flow application. (**C**) Average current-voltage (I–V) relationships in the absence and presence of fluid flow (n = 8); *p < 0.05, **p < 0.01, ***p < 0.001 vs control. (**D**) Fluid-flow-induced percentage increase in current at different voltage steps; n.s., no significant difference between the indicated voltages (as assessed by one-way analysis of variance [ANOVA]).

The IRK1 (IKir2.1) channel was reported to mediate a robust inward rectifier K^+^ current in RBL-2H3 cells^22^. We also confirmed the robust expression of Kir2.1 in RBL-2H3 cells by performing western blotting and immunocytochemistry experiments (Supplementary Fig. 1). Therefore, the RBL-2H3 cell line was an adequate model for studying the characteristics of IKir2.1. The IKir2.1 in these cells was elicited by using voltage steps up to −100 mV starting from a holding potential of 0 mV in the presence of a high-extracellular-K^+^ solution (148.4 mM KCl). The recorded currents were almost completely inhibited by BaCl_2_ (100 μM; Supplementary Fig. 2), which indicated that the currents were largely through Kir channels. Figure 1A shows representative IKir2.1 recordings under control (static) and fluid-flow conditions. Fluid flow increased the IKir2.1 by 10.3% ± 0.6% at −100 mV. This result is similar to that of Hoger *et al*.^18^. Figure 1C presents a summary of the current-voltage (I–V) relationships in the absence and presence of fluid flow. To obtain the I–V relationship, we applied voltage steps from −100 to +20 mV. The shape of the voltage-steps is shown as the figure inset. Figure 1B shows representative tracings of IKir2.1 at −100 mV before and after fluid-flow application. The onset of fluid flow immediately increased the IKir2.1. Fluid flow increased not only the inward component of the IKir2.1, but also its outward component (Fig. 1A,C,D). The percentage increases of IKir2.1 at various membrane potentials are summarized in Fig. 1D. The application of BaCl_2_ (100 μM) not only suppressed the majority of the currents, but also prevented the facilitating effect of fluid flow on the recorded currents (Supplementary Fig. 2)

### Effects of inhibitors of various kinases on the fluid flow-induced increase of IKir2.1

**Figure 2:**
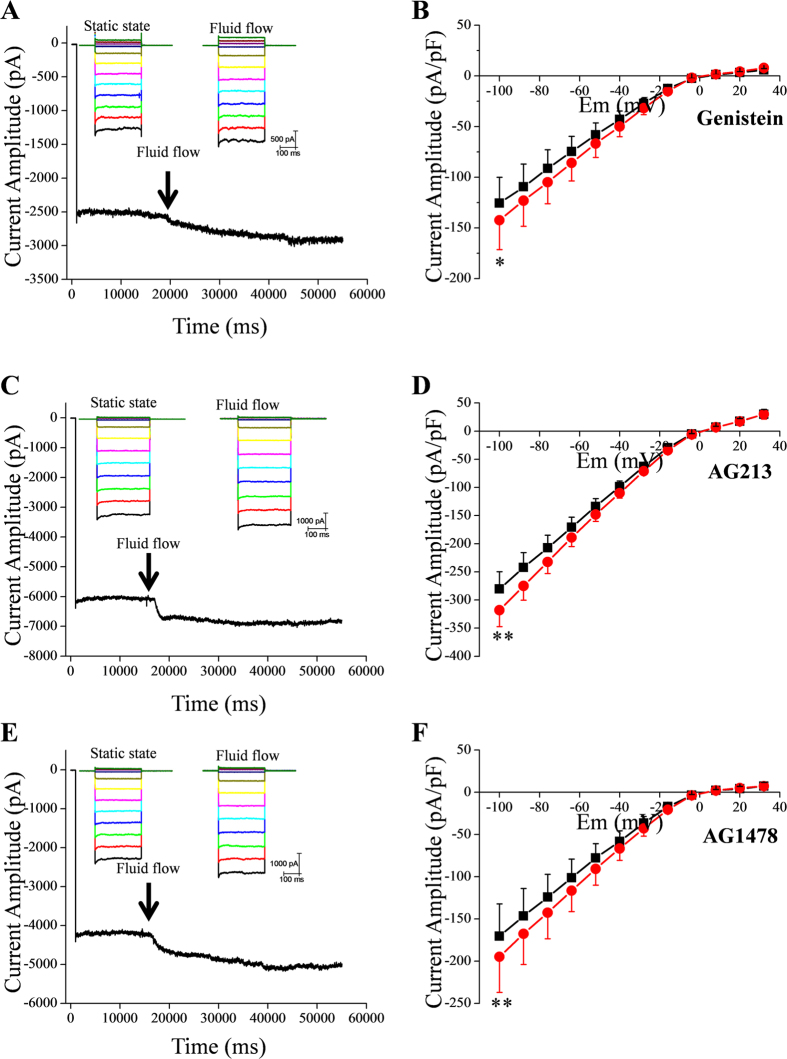
Effect of tyrosine kinase inhibitors on fluid flow-induced increase of IKir2.1. (**A,C**,**E**) Representative tracings of IKir2.1 at −100 mV before and after fluid-flow application in the presence of genistein (100 μM, **A**), AG213 (50 μM, **C**), and AG1478 (1 μM, **E**). The kinase inhibitors were included in the pipette solution. (**B**,**D**,**F**) Summary of the I–V relationships in the absence and presence of fluid flow after pretreatment with genistein, AG213, and AG1478 (n = 6, 5, and 4), respectively; *p < 0.05, **p < 0.01 vs control.

We investigated whether protein tyrosine kinases or serine/threonine kinases were involved in the fluid flow-induced increase of IKir2.1; we examined the effect of fluid flow on the IKir2.1 by using patch pipettes containing various kinase inhibitors. Fluid flow increased the recorded IKir2.1 even when we used pipettes containing genistein (100 μM), AG213 (50 μM), or AG1478 (1 μM), which are inhibitors of tyrosine kinases, PDGFR tyrosine kinase, and EGFR tyrosine kinase, respectively (Fig. 2). These results indicate that non-receptor and receptor tyrosine kinases did not mediate the increase of IKir2.1 induced by fluid flow. Furthermore, inclusion in the pipette of broad-spectrum serine/threonine kinase inhibitors, H7 (50 μM) or staurosporine (100 nM), also did not prevent fluid flow-induced increase of IKir2.1 (Supplementary Fig. 3). Staurosporine has been reported to inhibit various kinases such as PKC, PKA, Src tyrosine kinase, and Ca^2+^/calmodulin-dependent protein kinase^23^,^24^,^25^. Moreover, a cocktail of all the tested kinase inhibitors (genistein, AG213, AG1478, H7, and staurosporine) failed to prevent the increase of IKir2.1 induced by fluid flow (Supplementary Fig. 4), which suggests that phosphorylation was not involved in the fluid flow-induced activation of Kir2.1 channels.

To make sure that the kinase inhibitors used above worked properly in the present experimental conditions, we performed some positive control experiments under similar conditions. The results shown in Supplementary Figs 5 and 6 verify that the kinase inhibitors worked properly in this study.

### Effects of cytochalasin D, phalloidin, and methyl-β-cyclodextrin (MβCD)

**Figure 3:**
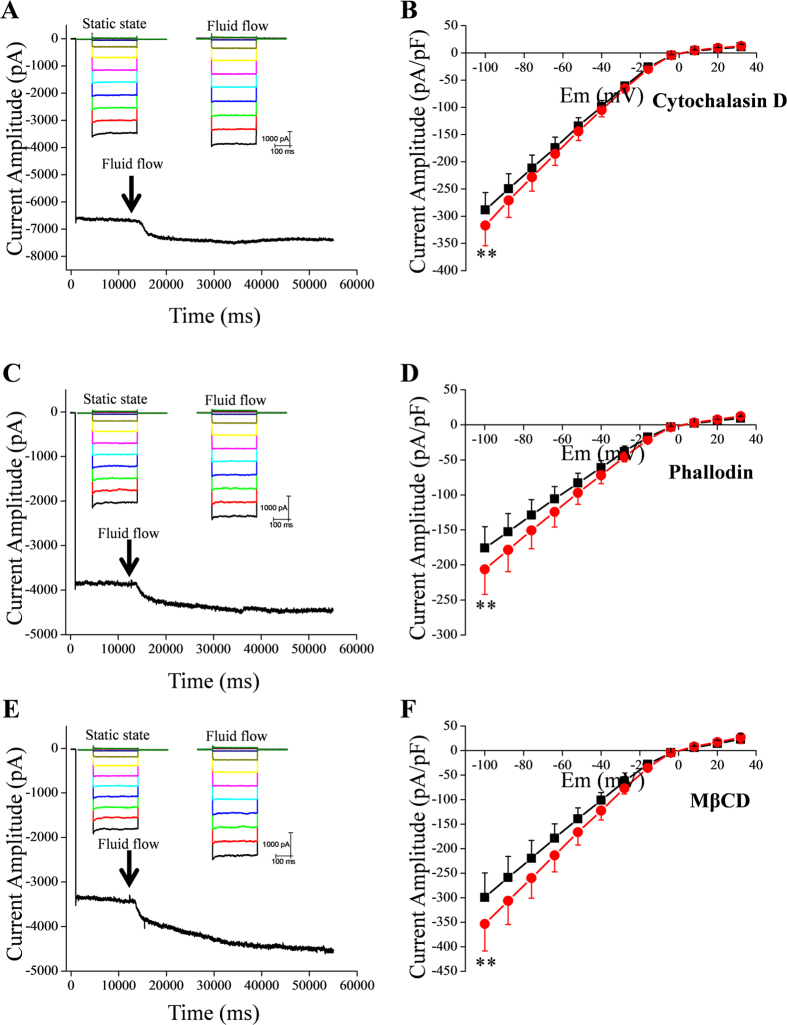
Effects of actin cytoskeleton disruption, stabilization of actin polymers, and disruption of caveolae on fluid flow-induced increase of IKir2.1. (**A**,**C**,**E**) Representative tracings of IKir2.1 at −100 mV before and after fluid-flow application, recorded from cells pretreated with cytochalasin D (**A**), phalloidin (**C**), and MβCD (**E**). (**B**,**D**,**F**) Summary of the I–V relationships in the absence and presence of fluid flow after pretreatment of cells with cytochalasin D (**B**), phalloidin (**D**), and MβCD (**F**) (n = 8, 8, and 6, respectively); **p < 0.01 vs control.

**Figure 4:**
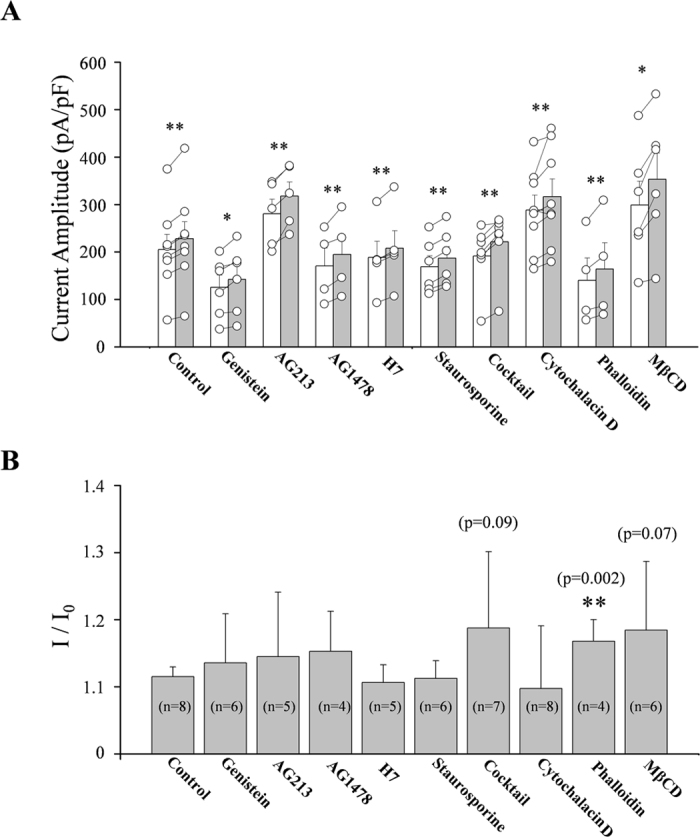
Summary of fluid-flow effects on IKir2.1 under various conditions. (**A**) Summary of the IKir2.1 amplitude under static and fluid-flow conditions in the absence and presence of various kinase inhibitors or cytoskeleton modulators. Open bars indicate static control and gray bars indicate fluid flow. Open circles indicate individual points from individual cells. The points from each individual cell under control and flow condition were connected with lines. (**B**) Summary of the ratio of fluid flow-induced IKir2.1 increase in the absence and presence of various kinase inhibitors or cytoskeleton modulators. Numbers in parenthesis in each bar indicate the number of cells examined. *p < 0.05, **p < 0.01 vs control.

Next, we examined whether the cytoskeleton was involved in the increase of IKir2.1 induced by fluid flow. Pipette application of cytochalasin D (20 μM), a disruptor of the actin cytoskeleton, or phalloidin, a stabilizer of actin polymers, did not prevent fluid flow-induced increase of IKir2.1 (Fig. 3). A recent study conducted by Kozera *et al*.^26^ suggested that caveolae function as a reservoir for mechanical stress and limit the stretch-activation of ion channels in ventricular myocytes. To test whether caveolae were involved in the fluid flow-dependent modulation of Kir2.1 channels, we examined the effect of MβCD, which disrupts caveolae. Our results showed that the disruption of caveolae caused by MβCD treatment (30 min) did not prevent the increase of IKir2.1 induced by fluid flow. In Fig. 4A, the increase of IKir2.1 under various conditions is summarized. Fluid flow increased the IKir2.1 by 10.3% ± 0.6% under control conditions, and this increase of IKir2.1 was more evident (16.8% ± 1.6%; p < 0.05) in cells pretreated with phalloidin (Fig. 4B). Interestingly, MβCD treatment also tended to strengthen the fluid-flow effect on the IKir2.1 (Fig. 4A,B), although the effect was not statistically significant (p = 0.07). We address this point further in the discussion section.

Again, to verify that the cytoskeletal and caveolar modifiers worked properly, we performed some positive control experiments. The results shown in Supplementary Figs 7 and 8 verified that the cytoskeletal and caveolar modifiers worked properly in this study.

### Simulation of the K^+^ ion concentration at the surface of the cell membrane during K^+^ influx by using the Nernst-Planck mass equation

The results described in the preceding subsections indicate that the increase of IKir2.1 induced by fluid flow does not require protein phosphorylation or cytoskeletal rearrangement. On the basis of these “negative results,” we examined the hypothesis that fluid flow might increase the IKir2.1 by restoring the original [K^+^] adjacent to the inlet (or outer mouth) of the Kir2.1 channel conduction pore, where [K^+^] falls below the average [K^+^] of the bulk extracellular solution during “rapid” K^+^ influx. This phenomenon might occur when the drop in [K^+^] at the Kir2.1 channel inlet during the “rapid” diffusion or influx of K^+^ across the cell membrane along its electrochemical gradient is not adequately restored (or equilibrated) solely by the diffusion or migration of the ion from the bulk extracellular solution. In this case, fluid flow might serve as convective transporter of K^+^ ions between the cell surface microdomain (channel inlet or outer mouth) and the bulk extracellular solution. As noted in the introduction section, Park *et al*.^10^ reported that for the movement of ions in fluids where an electrical field is applied, the contribution of the convection provided by fluid flow is >1,000 times larger than that of diffusion (ion movement induced by a concentration gradient) or migration (ion movement driven by an electrical gradient).

**Figure 5:**
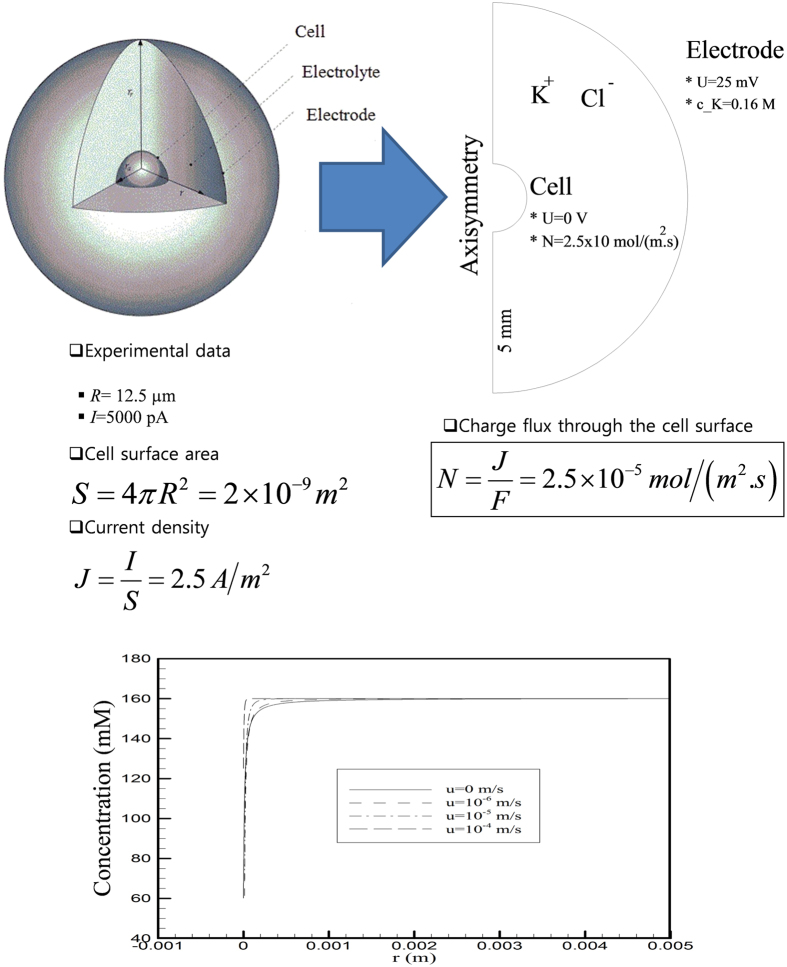
Schematic illustration of the simulation condition. (**A**) Simulations were performed using an artificial cell whose diameter, surface capacitance, and membrane conductance were assumed to be similar to those of RBL-2H3 cells: diameter = 12.5 μm; IKir2.1 density = 2.5 A/m^2^. (**B**) Simulation-derived relationship between [K^+^] and the distance from the cell surface. Abbreviations: U, electric potential; R, cell radius; I, total current; S, cell surface area; J, current density; N, charge flux; F, Faraday constant (F = 96,485 C mol^-1^).

In order to address above hypothesis, we performed simulations by using an artificial cell whose diameter and membrane conductance were assumed to be similar to those of RBL-2H3 cells. The extracellular solution domain was considered to represent a substantially larger sphere surrounding the artificial cell (Fig. 5A). According to the Nernst-Planck mass-flux equation listed below, we simulated a condition in which K^+^ is transported across the cell membrane from the extracellular domain to the cell interior in the absence and presence of extracellular fluid flow at various flow rates:


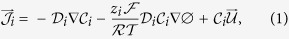


where *J*_*i*_ denotes the mass flux vector of species *i* (mol^−2^ s^−1^), c_*i*_ is the concentration (mol^−3^), D_*i*_ is its diffusion coefficient (m^2^ s^−1^), u is the velocity (m s^−1^), F is Faraday’s constant (96,485 C mol^−1^), R is the gas constant (8.314510 J K^−1^ mol^−1^), *ϕ* is the electric potential (V), and z the valence of the ionic species.The variables used in the simulation are shown in Fig. 5. In Fig. 5B, we present results summarizing the concentration gradient of K^+^ ions during K^+^ influx in the absence and presence of fluid flow. The results indicate that [K^+^] at the surface of the cell membrane might be markedly decreased during K^+^ influx, and further that fluid flow can restore the original [K^+^].

### Extracellular [K^+^]-Kir2.1 channel conductance ([K^+^]_o_-G_Kir2.1_) relationship

**Figure 6:**
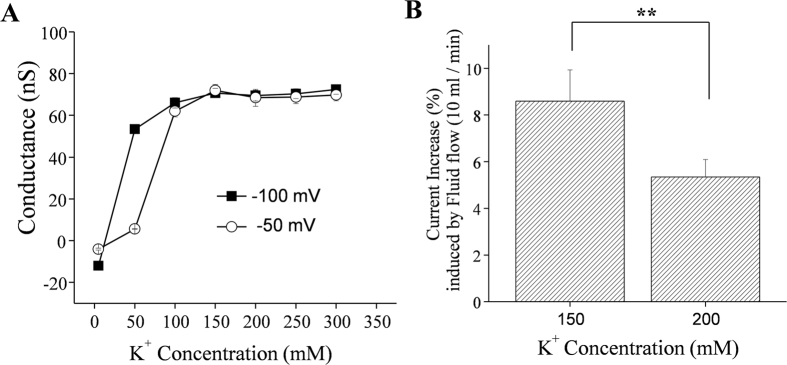
(**A**) Kir2.1 channel conductance-[K^+^] relationship. The conductance-[K^+^] relationship was obtained under the fluid-flow condition. Note the right-shifted conductance-[K^+^] relationship at −50 mV compared to the corresponding relationship at −100 mV. (**B**) Summary of the facilitating effect of fluid flow on the IKir2.1 at 150 and 200 mM extracellular [K^+^] at a voltage of −100 mV; **p < 0.01 vs control (n = 8 and 7).

The aforementioned simulation results suggest that the “effective” or “true” [K^+^] at the cell surface could fall below 2/3 of the average [K^+^] of the bulk extracellular solution. We reasoned that if the Kir2.1 channel conductance (G_Kir2.1_) becomes saturated as [K^+^]_o_ increases, the facilitating effect of fluid flow on IKir2.1 would be weakened at high extracellular [K^+^]. To test this hypothesis, we analyzed the G_Kir2.1_-[K^+^]_o_ relationship. As summarized in Fig. 6A, G_Kir2.1_ increased steeply as [K^+^]_o_ increased and saturated above a concentration of ~150 mM [K^+^]_o_. Furthermore, the G_Kir2.1_-[K^+^]_o_ relationship was found to be shifted to the right at a voltage of −50 mV compared with the corresponding relationship at −100 mV. The data in Fig. 6A were obtained under flow conditions. According to our simulation results, at [K^+^]_o_ of 150 mM, the effective or true [K^+^] near the cell surface would fall below 100 mM and fluid flow would restore this decrease in [K^+^] to distinct degrees depending on the fluid flow velocity. Thus, we would expect the degree of fluid flow-dependent facilitation of IKir2.1 to be lesser at higher (≥200 mM) [K^+^]_o_ than at lower (≤150 mM) [K^+^]_o_, because the [K^+^]_o_-G_Kir2.1_ relationship was saturated above 150 mM [K^+^]_o_ (Fig. 6A). In accord with this notion, the degree of flow-dependent facilitation of IKir2.1 was 8.6% ± 1.3% at 150 mM [K^+^]_o_ as compared with 5.3% ± 0.8% at 200 mM [K^+^]_o_ (Fig. 6B), which again supports the convection-regulation theory.

### Effects of fluid flow on the IKir2.1 in HEK293T cells ectopically over-expressing Kir2.1 channels

**Figure 7:**
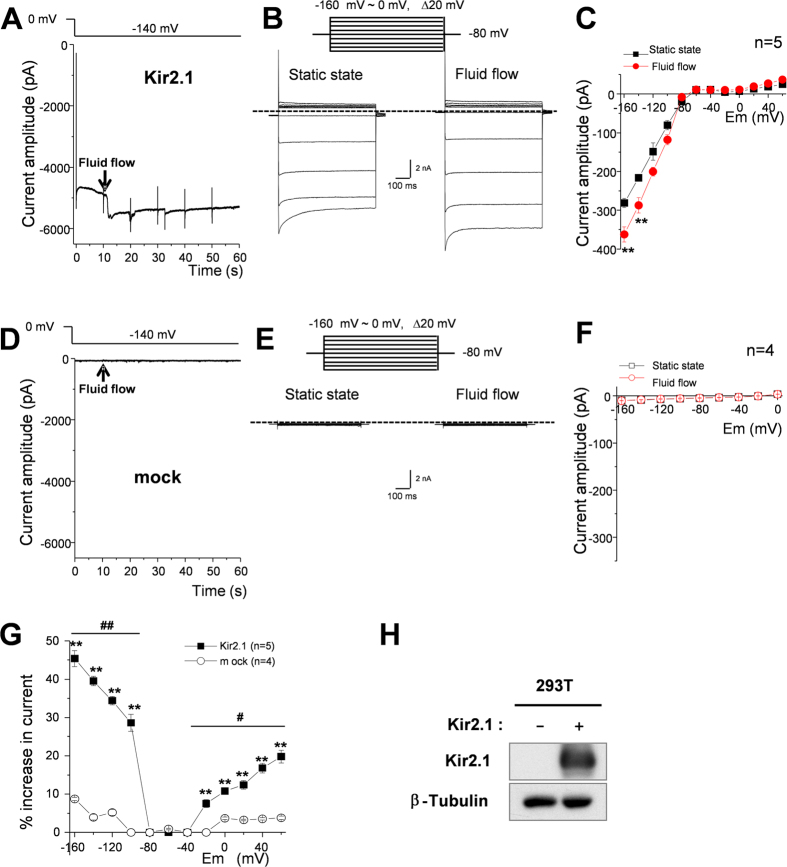
Kir2.1 activity is increased by fluid-flow in HEK293T cells in which Kir2.1 was ectopically over-expressed. (**A**,**D**) Representative tracing of currents at −140 mV in HEK293T cells expressing Kir2.1 (**A**) and non-transfected cells (**D**) before and after fluid-flow application. (**B**,**E**) Representative current recordings of HEK293T cells expressing Kir2.1 (**B**) and non-transfected cells (**E**) under control (static) and fluid-flow conditions. Currents were elicited by voltage steps from −160 mV to 0 mV at a holding potential of −80 mV. (**C**,**F**) Average current-voltage (I–V) relationships in the absence (black) and presence (red) of fluid flow in HEK293T cells expressing Kir2.1 (**C**) and non-transfected cells (**F**); **p < 0.01, static state vs fluid flow by Student’s *t*-test. (**G**) Fluid-flow-induced percentage increase in current at different voltage steps of HEK293T cells expressing Kir2.1 (closed symbol) and non-transfected cells (open symbol). **p < 0.01, Kir2.1 expression vs mock by Student’s *t*-test, ^#^p < 0.05, ^##^p < 0.01 between voltages by one-way ANOVA. (**H**) Western blot analysis to show Kir2.1 levels in HEK293T cells expressing Kir2.1 and non-transfected cells.

According to the simulation results in Fig. 5 and the data in Fig. 6, it is expected that the facilitating effect of fluid flow would be even larger at physiological low extracellular [K^+^] than at high extracellular [K^+^]. To examine this possibility, we decided to test the effect of fluid flow on the IKir2.1 at a physiological concentration of extracellular [K^+^] (i.e., 5.4 mM). To increase the ratio of IKir2.1 over other non-specific ion currents (i.e., the signal/noise ratio) under low extracellular [K^+^], we used HEK293T cells that were transfected to ectopically over-express mouse Kir2.1 channels instead of RBL2H3 cells. Figure 7B shows representative IKir2.1 recordings at various voltages in HEK293T cells overexpressing Kir2.1 channels under control (static) and fluid-flow conditions. Figure 7A shows representative tracings of IKir2.1 at −140 mV before and after fluid-flow application. The onset of fluid flow immediately increased the IKir2.1 in HEK293T cells. Figure 1C presents a summary of the I-V relationships in the absence and presence of fluid flow. The I–V curves in the absence and presence of fluid flow crossed each other at a voltage between −80 and −100 mV, indicating that the augmentation of current by fluid flow was K^+^ selective. Figure 7D–F show the negative controls from mock-treated non-transfected HEK293T cells. No IKir2.1 and no effect of fluid flow are evident. Figure 7H again shows the selective expression of Kir2.1 in the transfected HEK293T cells compared with the non-transfected mock control by western blotting. Finally, Fig. 1G summarizes the increasing effect of fluid flow on the IKir2.1 at different voltages. In accordance to our hypothesis, the increase of IKir2.1 by fluid flow was even larger and clearly voltage-dependent (driving force-dependent) when the extracellular [K^+^] was low (5.4 mM; compare Fig. 7G with Fig. 1D, where IKir2.1 was recorded under a high extracellular [K^+^] of 148.4 mM).

### Effect of fluid flow on the E_m_ of RBL2H3 cells

**Figure 8:**
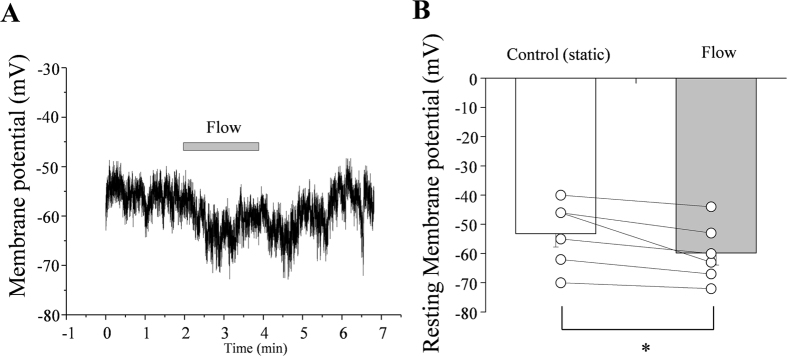
Effect of fluid flow on the membrane potential (E_m_) of RBL2H3 cells. (**A**) Representative tracing of E_m_ showing the effect of fluid flow. (**B**) Summary of E_m_ values in the absence and presence of fluid flow. Open circles indicate individual points from individual cells. The points from each individual cell under control and flow condition were connected with lines. *p < 0.05 (n = 6).

Finally, we examined whether fluid flow can hyperpolarize the E_m_ of RBL2H3 cells. The outward component of IKir2.1 is physiologically relevant in most cases, and fluid flow increased both the inward and outward components of IKir2.1 (Figs 1D and 7G). In accordance with the above results, the application of fluid flow hyperpolarized the E_m_ of RBL2H3 cells (Fig. 8) by ~6.7 mV (from 53.1 ± 4.6 mV to 59.8 ± 4.1 mV, n = 6) in a reversible manner.

## Discussion

On a warm spring day, a boy playing in a playground experiences a pleasant sensation when a breeze touches his face. What arouses this pleasant feeling? Is it the gentle touch of the breeze on the skin of the boy’s face, or the temperature decrease near the skin? Whereas the effect of the breeze’s touch involves the mechanotransduction of shear force (air flow) into tactile sensation, the temperature change involves merely convective refreshment of the air near the skin, which results in a facilitated reduction in skin temperature. In most cases, the temperature decrease is likely to contribute comparatively more to the pleasant sensation, because conduction of heat from the body surface to the air is slow and the air convection caused by the breeze strongly facilitates the emission of heat generated during physical activity (the boy playing). We propose that the facilitation of IKir2.1 by fluid flow reported herein is similar to the aforementioned case: In this study, we demonstrated that fluid flow increased the IKir2.1 in RBL-2H3 cells. This fluid flow-induced facilitation of IKir2.1 was independent of protein phosphorylation and cytoskeleton rearrangement. Moreover, simulations performed using an artificial cell–whose membrane properties were similar to those of RBL-2H3 cells–suggested that fluid flow could increase the IKir2.1 through convective transport of K^+^ to the Kir2.1 channel inlet (or outer mouth), where [K^+^] during K^+^ influx is considerably lower than the average [K^+^] of the bulk extracellular solution.

Kir2.1 channel activity was reported to be increased immediately following the application of shear stress in a heterologous expression system^18^. The fluid flow-induced increase of IKir2.1 was regulated by tyrosine kinases. However, our results indicate that the fluid flow-induced increase of IKir2.1 in RBL-2H3 cells was not mediated by tyrosine kinases (Fig. 2). The reason for this discrepancy is not certain, although the differences in cells or the experimental system could be partly responsible. However, one clear-cut result obtained was that fluid flow increased the IKir2.1 in RBL-2H3 cells even in the absence of kinase activity: treatment of cells with a cocktail of various potent inhibitors of serine/threonine and tyrosine kinases did not prevent fluid flow-induced facilitation of Kir2.1 (Supplementary Figs 3 and 4). Kir2.x channels contain several tyrosine and serine/threonine residues, and Kir2.3 was reported to be facilitated by phosphorylation at Y234^14^,^15^,^16^,^17^,^27^,^28^ Moreover, tyrosine phosphorylation of Kir2.1 (at Y242 and Y366) was reported to mediate the channel’s activation by shear force^18^. The contributions of these Kir2.1 phosphorylation sites in fluid flow-induced modulation of the channel in RBL-2H3 cells warrant further investigation; nevertheless, our results suggest a novel mechanism for the fluid flow-induced facilitation of Kir channels. We believe that the “fluid flow-convection hypothesis” of this study could be applied to any ion channel whose single-channel conductance is “sufficiently large” and open time is “sufficiently long.”, which are reasoned to be the required properties for the accumulation of ions near the channel mouth.

Fluid flow has been reported to regulate ion channel activity through cytoskeleton modulation or morphology change depending on the exposure time^29^. Therefore, we examined how channel activity was affected by the pretreatment of cells with cytochalasin D, which disrupts the actin cytoskeleton, or phalloidin, which stabilizes actin polymers^30^,^31^,^32^, and we found that these reagents did not prevent the increase of IKir2.1 induced by fluid flow (Figs 3 and 4). Caveolae are omega-shaped cholesterol-rich regions of the cell membrane that have been reported to mediate numerous receptor-mediated or mechanical stress-mediated responses^33^,^34^. Therefore, we examined the effect of MβCD on fluid flow-induced IKir2.1 facilitation: MβCD has been reported to reduce the number of caveolae by depleting cholesterol in the membrane. We found that MβCD treatment reduced the cell surface membrane, which was reflected as a lowering of membrane capacitance (control vs after MβCD treatment: 20.8 ± 0.9 vs 14.27 ± 0.8 pF); this indicated that RBL-2H3 cells contain numerous caveolae under control conditions. However, the disruption of caveolae did not prevent the fluid flow-induced facilitation of IKir2.1. Intriguingly, the IKir2.1 increase induced by fluid flow was markedly larger in cells pretreated with phalloidin than in control cells (Fig. 1). Furthermore, although the effect was not statistically significant (p = 0.07), pretreatment with MβCD also tended to increase the “degree of IKir2.1 increase by fluid flow”. This could be interpreted as follows: certain structures related to the cytoskeleton, such as the omega-shaped caveolae, are sequestrated from the effects of fluid flow because of the membrane geometry, and this could decrease the convection-mediated facilitating effect of fluid flow on IKir2.1. Disruption of caveolae exposes the sequestrated membrane region, which results in the enhanced increase of IKir2.1 by fluid flow. A comparison of the caveolar structure to *strait* or *sound* in geography suggests that our findings might be similar to what is observed in the case of a *strait* or *sound*: Although seawater fills the space in a strait or sound, the composition of the “sea water” in the strait or sound is similar to that of freshwater, and the steeper the geometry of the strait or sound is, the closer to freshwater the composition of seawater is. This is because the water in the strait or sound is sequestrated from the convective tides of the sea.

Here, we have suggested for the first time that the effective concentration of an ion can differ considerably between the channel inlet microdomain and the bulk extracellular solution during ion current flux. This phenomenon might be observed when the single-channel conductance of an ion channel is sufficiently large and the diffusion between the bulk phase and the microdomain (channel inlet or outer mouth of channel) is not adequately rapid. In such a scenario, fluid flow is the dominant mode of ion movement in the solution, and this flow increases the ion current by elevating the effective concentration of ions at the channel inlet. By performing simulations on artificial cells whose diameter and membrane conductance were assumed to be similar to those of RBL-2H3 cells, we indirectly showed that a concentration gradient must exist between the surface microdomain and the bulk domain. Comparing the magnitudes of the increase in the current under different voltages further supported the “convection-regulation hypothesis”. According to this hypothesis, at more hyperpolarized voltages relative to the K^+^ equilibrium potential, the magnitude of change in the current should be bigger, because the larger driving force moves the K^+^ ions into the cell more quickly and depletes the local extracellular ions more completely. This was exactly what was observed in the data presented in Fig. 7; in particular, Fig. 7G shows that the magnitudes of the increases in the current were much larger at more hyperpolarized potentials. This hypothesis also applies to the outward component of the IKir2.1. At more depolarized voltages relative to the K^+^ equilibrium potential, the magnitude of the change in the current should be bigger, because the larger driving force moves the K^+^ ions out of the cell more quickly and accumulates local extracellular ions more. Again, this was also confirmed by the experimental results (Fig. 7G). Moreover, the G_Kir2.1_-[K^+^]_o_ relationship shown in Fig. 6 also supports the “convection-regulation hypothesis”. At −100 mV, compared with −50 mV, the saturation point was found at a lower [K^+^]_o_ (note that the voltage of −100 mV provides a larger driving force than −50 mV in Fig. 6). These observations confirmed that the magnitude of change in the current correlates with the amount of K^+^ movement. In the experiments for Fig. 1D, where a high concentration of KCl bath solution was used, these phenomena were not found to be significant. This might be because the current amplitudes (or conductance of Kir2.1) were already near to a saturating level at a high (148.4 mM) [K^+^]_out_ (see Fig. 6) and the depletion/accumulation of local K^+^ ions at the channel outer mouth were near saturation to the “convection-regulation”.

Intriguingly, the degree of inactivation (which was voltage and time dependent) observed at hyperpolarized potentials was slightly decreased by fluid flow (raw traces in Figs 1, 2, 3, 7 and Supplementary Figs 3 and 4). We interpret these data to mean that the initial inactivation of Kir2.1 was also partly contributed by the lowered [K^+^] at the channel inlet, because fluid flow partly decreased the inactivation (Figs 1, 2, 3, 7 and Supplementary Figs 3 and 4). The mechanism of this time- and voltage-dependent inactivation of Kir2.1 channels warrants further investigation.

In conclusion, we suggest here for the first time that fluid flow might increase IKir2.1 by elevating effective [K^+^] at the channel inlet, where [K^+^] decreases during current flux. This novel interpretation of fluid flow-induced ion channel regulation should be considered when analyzing the effect of fluid flow-induced shear stress on ion channels as well as the inactivation kinetics of ion channels.

## Materials and Methods

### Cell culture

We cultured the RBL-2H3 cell line (ATCC^®^ CRL-2256™) in MEM (minimum essential medium; Invitrogen, 11095, Waltham, MA USA) supplemented with 15% FBS (fetal bovine serum; Hyclone, Logan, Utah) and 1% penicillin/streptomycin (Invitrogen) and maintained the cells in a 5% CO_2_ balance incubator.

### Western blotting

RBL-2H3 cells were grown to 80% confluence and starved in MEM without FBS for 12 h before experiments. The cells were then washed twice with PBS and lysed using RIPA buffer (TNT Research, Seoul, South Korea). Samples were run on a 10% SDS-polyacrylamide non-reducing gel and then transferred to a polyvinylidene fluoride membrane (Millipore, Bedford, MA, USA), and the membrane was blocked with 5% BSA for 1 h. Western blotting was performed using rabbit primary antibodies against Kir2.1 channel (1:500; Alomone Lab, Jerusalem, Israel) and secondary antibodies conjugated with horseradish peroxidase (1:1,000; Cell Signaling Technology, Danvers, MA, USA). For the negative control, the Kir2.1 channel antibody was incubated with the Kir2.1 antigen at room temperature for 1 h before blotting. Signals were visualized using the Las-4000 system (Fuji Film, Tokyo, Japan).

To perform western blot analysis with Kir2.1-expressing HEK293T cells, the cells were lysed with a lysis buffer composed of 10 mM Tris-HCl (pH 8.0), 150 mM NaCl, 1 mM ethylenediaminetetraacetic acid, 1% Triton™ X-100, and complete protease inhibitor cocktail (Roche Diagnostics, Indianapolis, IN). Then, the prepared samples were separated by 10% SDS-polyacrylamide gel electrophoresis. After transferring to a polyvinylidene fluoride membrane (Immobilon-P; Millipore, Billerica, MA), the membrane was blotted with anti-Kir2.1 antibody (1:500; Santa Cruz Biotechnology, Santa Cruz, CA) and anti-β-tubulin antibody (1:1000; Santa Cruz Biotechnology).

### Immunocytochemistry

RBL-2H3 cells were cultured on LAB-TEK (Thermo Fisher Scientific, Waltham, MA USA) and then treated with 4% paraformaldehyde for 30 min and 0.1% Triton X-100 for 10 min and blocked with 5% BSA for 1 h. Next, the cells were incubated overnight at 4 °C with rabbit primary antibodies against Kir2.1 channel (1:100; Alomone Lab, Jerusalem, Israel). Cy2-conjugated secondary antibodies were used in the immunocytochemistry (1:1,000; Alomone Lab), and nuclei were stained (1 h) withTO-PRO-3 (Thermo Fisher Scientific, Waltham, MA USA). The stained RBL-2H3 cells were examined under a confocal microscope (ZEISS LMS-710, Oberkochen, Germany).

### Electrophysiology and application of fluid flow

The inwardly rectifying K^+^ current of RBL-2H3 cell was recorded using a whole-cell patch-clamp configuration^35^ and an EPC 8 patch-clamp amplifier (Heka, Lambrecht/Pfalz, Germany). Data were digitized using custom-built software (R-clamp; provided by Dr. S.Y. Ryu), at a sampling rate of 5 kHz, after being low-pass filtered at 1 kHz, and then stored on a computer. Voltage pulse generation was also controlled by R-clamp software. The patch pipettes were pulled from borosilicate capillaries (Clark Electromedical Instruments, Pangbourne, UK) by using a puller (PP-83; Narishige, Tokyo, Japan); we used patch pipettes that featured a resistance of 1.5–2.5 MΩ when filled with the pipette solution. All experiments were conducted at room temperature (20–24 °C). A commercial bathing chamber (RC-11, WPI, Sarasota, FL, USA) was used, and an Ag/AgCl pellet electrode (WPI, USA) in 3 M KCl and the bath solution were connected by a 3 M KCl agar-bridge. The fluid was applied using gravity flow (flow rate: 10 mL/min).

### Expression of Kir2.1 channels in HEK293T cells

To prepare HEK293T cells ectopically expressing Kir2.1, the cells were cultured in Dulbecco’s modified Eagle’s medium (Invitrogen, Carlsbad, CA) with 10% FBS and 1% penicillin/streptomycin, and transfected with a mouse Kir2.1 expression vector (pcDNA-HA-Kir2.1) and Lipofectamine^®^ 2000 (Invitrogen). The Kir2.1 expression vector was kindly provided by Dr. Penelope Jones (University of California, San Diego, CA).

### Solutions and drugs

The normal Tyrode solution (143 mM NaCl, 5.4 mM KCl, 0.33 mM NaH_2_PO_4_, 5 mM HEPES, 0.5 mM MgCl_2_, 1.8 mM CaCl_2_, 11 mM D-glucose; pH adjusted to 7.4 with NaOH) was used as the bathing solution during the establishment of the whole-cell mode. High-K^+^ Tyrode solution (148.4 mM KCl, 0.33 mM NaH_2_PO_4_, 5 mM HEPES, 0.5 mM MgCl_2_, 1.8 mM CaCl_2_, 11 mM D-glucose; pH adjusted to 7.4 with NaOH) was used as the bathing solution for examining Kir currents. The pipette solution contained 135 mM KCl, 5 mM NaCl, 5 mM Mg-ATP, 10 mM HEPES, 5 mM ethyleneglycol-bis (2-aminoethyl)-N,N,N′,N′,-tetraacetic acid (EGTA), pH 7.2 (adjusted with KOH). Because RBL-2H3 cells are highly susceptible to hypo-osmotic swelling and consequent activation of volume-activated Cl^−^ currents, 38 mM sucrose was added to the Tyrode solutions in order to adjust osmolarity and prevent cell swelling. Moreover, a Cl^−^ channel blocker, 4,4′-diisothiocyano-2,2′-stilbenedisulfonic acid (DIDS, 30 μM), was added to the pipette solution to eliminate any contamination by Cl^−^ currents. The composition of the 300 mM [K^+^] bath solutions used for the experiments shown in Fig. 6 was the following: 300 mM KCl, 0.33 mM NaH_2_PO_4_, 5 mM HEPES, 0.5 mM MgCl_2_, 1.8 mM CaCl_2_, 11 mM glucose, and 25 mM sucrose. The various [K^+^] bath solutions were prepared through equimolar substitution of KCl with NaCl. The pipette solution used for these experiments contained 300 mM KCl, 5 mM NaCl, 10 mM HEPES, 5 mM EGTA, 5 mM Mg-ATP, 30 μM DIDS, pH 7.2. All chemicals and drugs were purchased from Sigma-Aldrich (St. Louis, USA) and Tocris Bioscience (Bristol, UK).

### Statistical analysis

The results are shown as means ± S.E.M. Paired or independent Student’s *t*-tests and one-way ANOVA were used to test for significance; p < 0.05 was considered statistically significant.

## Additional Information

**How to cite this article**: Kim, J. G. *et al*. Fluid flow facilitates inward rectifier K^+^ current by convectively restoring [K^+^] at the cell membrane surface. *Sci. Rep.* **6**, 39585; doi: 10.1038/srep39585 (2016).

**Publisher's note:** Springer Nature remains neutral with regard to jurisdictional claims in published maps and institutional affiliations.

## Supplementary Material

Supplementary Information
