# Supplementary material for: Fluid flow facilitates inward rectifier K+ current by convectively restoring [K+] at the cell membrane surface
Source: Sci Rep. 2016 Dec 22;6:39585. doi: 10.1038/srep39585 (PMC5177964; doi:10.1038/srep39585)
Supplement: Supplementary Information [file srep39585-s1.pdf]

Supplementary information of the manuscript:

Fluid flow facilitates inward rectifier K^+ current by convectively restoring $[K^+]$ at the cell membrane surface

Jae Gon Kim[1], Sang Woong Park[2], Doyoung Byun[3], Wahn Soo Choi[4], Dong Jun Sung[5], Kyung Chul Shin[1], Hyun-ji Kim[6], Young-Eun Leem[7], Jong-Sun Kang[7], Hana Cho[6], Bokyung Kim[1], Sung Il Cho[1], and Young Min Bae[1*]

[1]*Department of Physiology, KU Open Innovation Center, Research Institute of Medical Science, Konkuk University School of Medicine, Chungju, Chungbuk 380-701, South Korea*
[2]*Department of Emergency Medical Services, Eulji University, Seongnam, Gyeonggi-do, 461-713, South Korea*
[3]*Department of Mechanical Engineering, Sungkyunkwan University, 2066 Seobu-Ro, Jangan-Gu, Suwon, Gyeonggi 440-746, South Korea.*
[4]*Department of Immunology, School of Medicine, Konkuk University, Chungju 380-701, South Korea*
[5]*Division of Sport Science, College of Science and Technology, Konkuk University, Chungju 380-701, South Korea*

[6]*Department of Physiology and Samsung Biomedical Research Institute, Sungkyunkwan University School of Medicine, Suwon 440-746, South Korea*

[7]*Department of Molecular Cell Biology and Samsung Biomedical Research Institute, Sungkyunkwan University School of Medicine, Suwon 440-746, South Korea*

**Corresponding author; E-mail: ymbae30@kku.ac.kr*

SI Materials and Methods

Intracellular $[Ca^{2+}]_i$ measurements

RBL2H3 cells were incubated with a concentration of 5 μM Fura-2AM (Thermo Fisher Scientific, Waltham, MA, USA) for 30 min at room temperature. The cells were then loaded to a chamber and washed in normal Tyrode's solution (NT) for 30 min at room temperature. For measurements with Fura-2AM, the excitation wavelengths were 340 and 380 nm and the emission wavelength was 510 nm (Lambda DG-4, Sutter Instrument Company, Novato, CA, USA). Cells were sensitized with anti-DNP IgE (200 ng/mL) for 4 h and stimulated with DNP-bovine serum albumin (BSA) (100 ng/mL).

A7R5 cells and recording of voltage-dependent Ca^{2+} channel currents

The A7r5 cell line, derived from embryonic rat aorta, was used for voltage-gated Ca^{2+} channel current recording. The cells were grown with high-glucose Dulbecco's modified Eagle's medium (DMEM) containing 10% fetal bovine serum and 1% penicillin-streptomycin. Before performing patch-clamp experiments, the cells were dispersed with 0.5% trypsin-ethylenediaminetetraacetic acid. For recording voltage-dependent Ca^{2+} channel currents, the whole cell patch pipette solution contained (in mM): CsCl, 120; $MgCl_2$, 1; 4-(2-hydroxyethyl)-1-piperazineethanesulfonic acid, 5; MgATP, 5; 1,2-*bis*(aminophenoxy)ethane-*N*,*N*,*N'*,*N'*-tetraacetic acid, 10, and the pH was adjusted to 7.2 by CsOH. The bath solution contained 10 mM $BaCl_2$ in Ca^{2+}-free NT solution.

SI Results

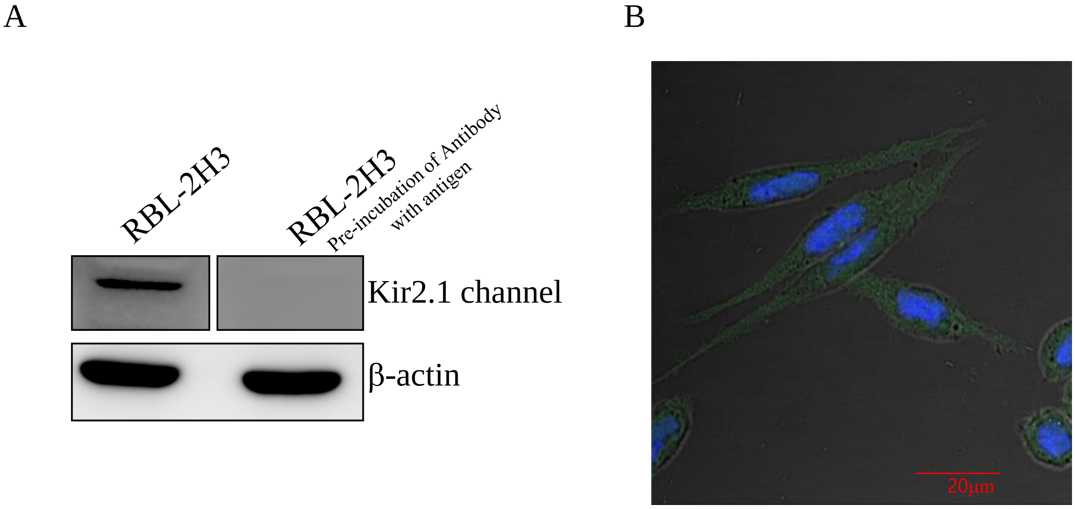

Supplementary Fig. 1. Expression of Kir 2.1 channel in RBL-2H3 cells. A, Representative western blotting result showing the robust expression of Kir2.1 in RBL-2H3 cells (left lane). For proving the specificity of Kir2.1 antibody, the antibody was incubated with Kir 2.1 channel antigen at room temperature for 1h before the blotting (right lane). The two lanes were from the same membrane that has been run under the same experimental procedure; for incubation with the mixture of Kir2.1 antigen and antibody, the right lane was cropped from the original membrane. B. Representative immunocytochemistry picture of Kir2.1 channel in RBL-2H3 cells. Green color indicates Kir2.1 channels and blue color indicates nucleus.

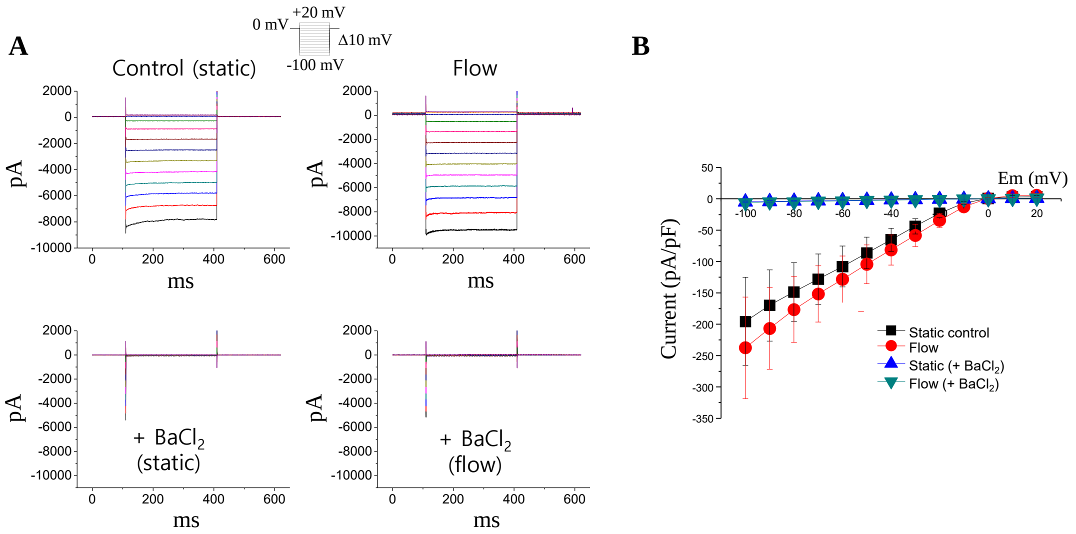

Supplementary Fig. 2. Effect of $BaCl_2$ (100 μM) on IKir2.1 in the absence and presence of fluid flow. A, Representative Kir2.1 currents elicited by voltage steps starting from a holding potential of 0 mV (the shape of which is shown as a figure inset), under static control (left) and fluid-flow (right) conditions. Upper panels, in the absence of $BaCl_2$. Lower panels, in the presence of $BaCl_2$. B, Summary of I-V relationships of IKir2.1 under each condition (n = 5).

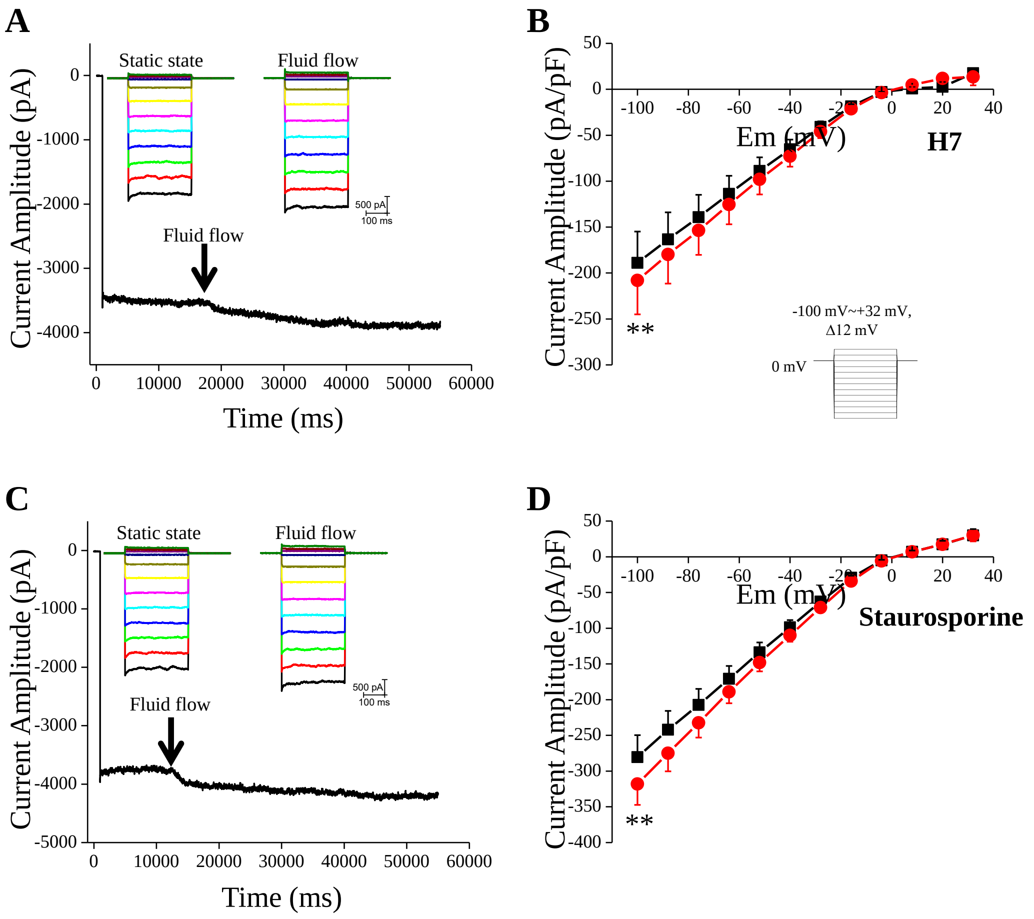

Supplementary Fig. 3. Effect of broad-spectrum serine/threonine kinase inhibitors on fluid flow-induced increase of IKir2.1. A and C. Representative tracings of IKir2.1 at -100 mV before and after fluid-flow application in the presence of H7 (50 μM) and staurosporine (100 nM). B and D. Summary of the I-V relationships in the absence and presence of fluid flow after pretreatment of RBL-2H3 cells with H7 and staurosporine (n = 5 and 6), respectively; $^{**}p < 0.01$ vs control.

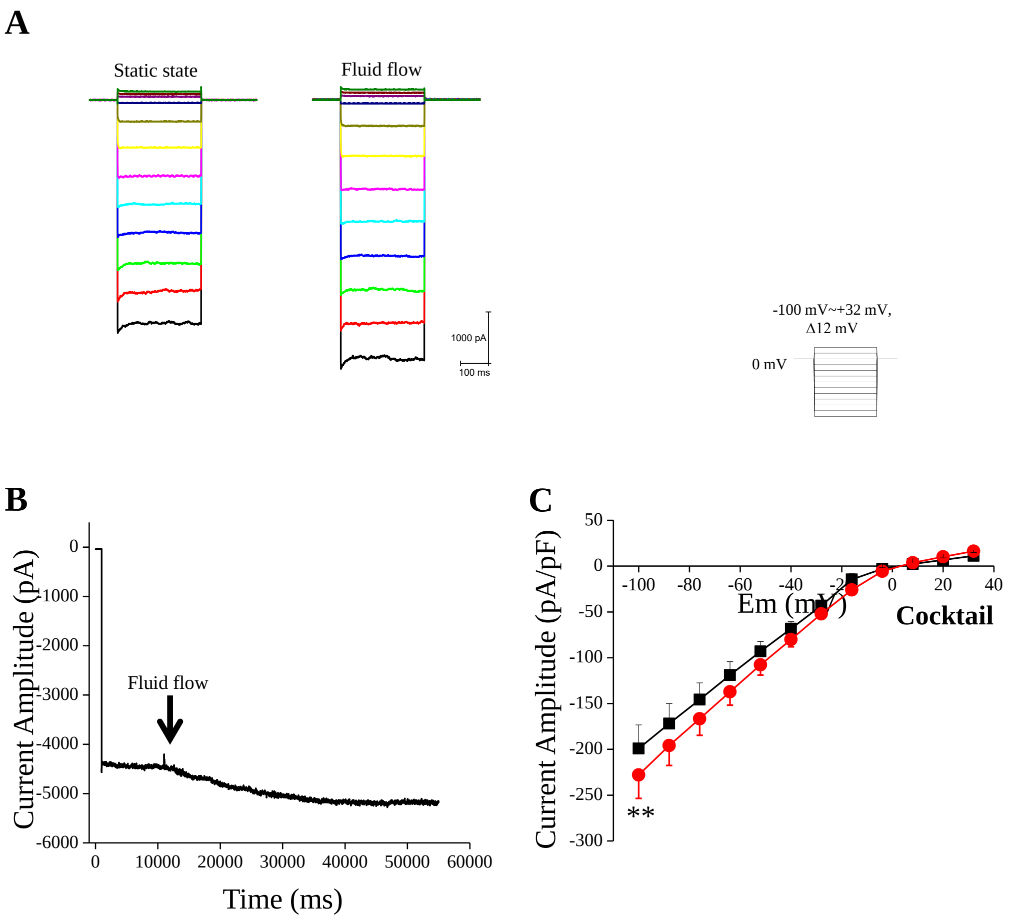

Supplementary Fig. 4. Effect of a cocktail of tyrosine kinase inhibitors and broad-spectrum serine/threonine kinase inhibitors on fluid flow-induced increase of IKir2.1. A. Representative Kir2.1 currents under control (static) and fluid-flow conditions, recorded from cells pretreated with the inhibitor cocktail. B. Representative tracing of IKir2.1 at -100 mV before and after fluid-flow application, recorded from a cell pretreated with the inhibitor cocktail. C. Summary of the I-V relationships in the absence and presence of fluid flow after pretreatment of RBL-2H3 cells with the inhibitor cocktail; n = 7, **p < 0.01 vs control.

In RBL2H3 cells, it was reported that protein kinase A (PKA)-sensitive volume-activated Cl^- currents spontaneously developed after whole cell formation[1]. In our present experiments, we also observed these spontaneously-developed Cl^- currents; hence, we added extracellular sucrose (38 mM) and pipette 4,4′-diisothiocyano-2,2′-stilbenedisulfonic acid (DIDS, 30 μM) to block these Cl^- currents (See *Material and Methods* section of main manuscript). In supplementary Fig. 5A, a time course of the gradual spontaneous development of Cl^- currents in the absence of extracellular sucrose and pipette DIDS is shown. These spontaneous currents were significantly inhibited by staurosporine application (100 nM, supplementary Fig. 5A). The I-V relationships that were obtained with step voltage pulses in the absence and presence of staurosporine (100 nM) in the pipette are compared in supplementary Fig. 5B.

It is well-known that RBL2H3 mast cells can be activated by antigen stimulation such as DNP-BSA after sensitization with anti-DNP IgE[2]. Upon stimulation, $[Ca^{2+}]_i$ increases and store-operated Ca^{2+} channels (SOC) are activated[2]. These responses are mediated through the activation of tyrosine kinases such as Lyn[2]. SOC can also be activated by thapsigargin, an endoplasmic Ca^{2+} pump inhibitor[2]. Supplementary Figs. 6A and 6B show that both the antigen-stimulated $[Ca^{2+}]_i$ increase and the activation of SOC by thapsigargin were suppressed by pretreatment with genistein (10 μM). These results indicate that staurosporine and genistein worked as expected and inhibited PKA and PTK, respectively, in this study.

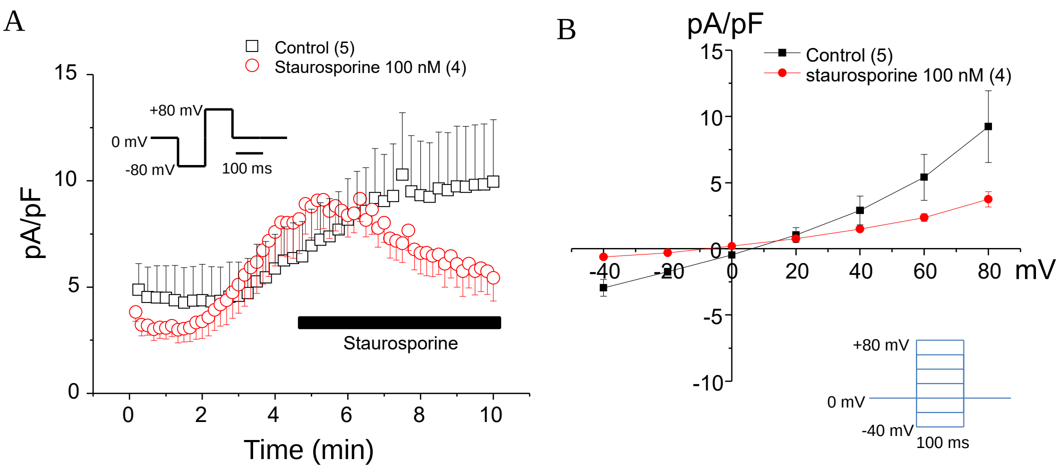

Supplementary Fig. 5. Effects of a protein kinase inhibitor, staurosporine, on the Cl¯ current in RBL2H3 cells. Cl¯ currents were recorded using the whole cell patch-clamp technique with a normal-high (135 mM) K^+ pipette and Tyrode's bath solution. **A**, Time-courses of the Cl¯ current development (recorded at +80 mV) after whole cell formation in the absence and presence of staurosporine are summarized. Black open squares indicate the time control of Cl¯ current development; in red open circles, the duration of staurosporine is indicated by a horizontal black bar. **B,** I-V relationships of the Cl¯ currents elicited by voltage steps in the absence and presence of staurosporine are summarized. Numbers in parentheses indicate the numbers of cells examined.

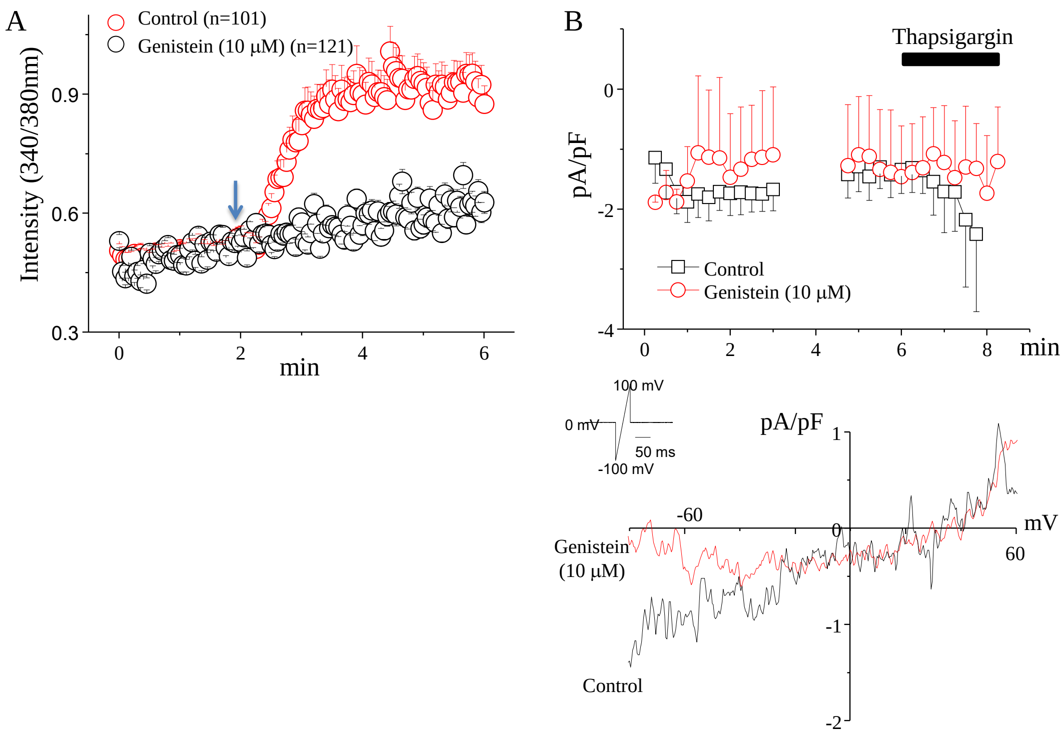

Supplementary Fig. 6. Effects of a protein tyrosine kinase (PTK) inhibitor (genistein) on antigen-stimulated Ca^{2+} increase and thapsigargin-induced SOC current in RBL2H3 cells. RBL2H3 cells were sensitized with anti-DNP IgE (200 ng/mL) for 4 h before antigen-stimulation. A, Summary of the antigen (DNP-BSA, 100 ng/mL)-stimulation effect on $[Ca^{2+}]_i$ in control and genistein-pretreated cells. Stimulation with antigen (indicated with blue arrow) markedly increased $[Ca^{2+}]_i$ in control cells, whereas it had little effect on $[Ca^{2+}]_i$ in the genistein-pretreated cells. B, Effect of genistein on the thapsigargin (2 μM)-induced SOC current development (at −80 mV) (control n = 4, genistein n = 4). C, Representative I-V relationships, which were obtained by subtracting current traces before and after thapsigargin treatment, are compared between control and genistein-pretreated cells. In B & C, the bath solution contained 10 mM Ca^{2+}, and 135 mM CsCl pipette solution (135 mM KCl pipette solution was substituted with equimolar CsCl solution) with or without genistein was used.

It was reported that actin disruption by cytochalasin D inhibited voltage-dependent Ca^{2+} channels in cardiomyocytes[3]. We could also observe this inhibitory effect of cytochalasin D on the voltage-dependent Ca^{2+} channels in A7R5 rat aortic smooth muscle cells (supplementary Fig. 7). Moreover, the application of phalloidin together with cytochalasin D in the pipette partly rescued the effect of cytochalasin D (supplementary Fig. 7), indicating that these chemicals properly worked as cytoskeleton modifiers in this study.

The caveolar modifier MβCD (a disruptor of caveolae) was previously reported to inhibit the Ca^{2+}-increase normally observed in mast cells upon antigen-stimulation[2]. We also clearly observed that pretreatment with MβCD prevented the antigen-induced $[Ca^{2+}]_i$ increase in RBL2H3 cells (supplementary Fig. 8), verifying that MβCD properly worked in this study.

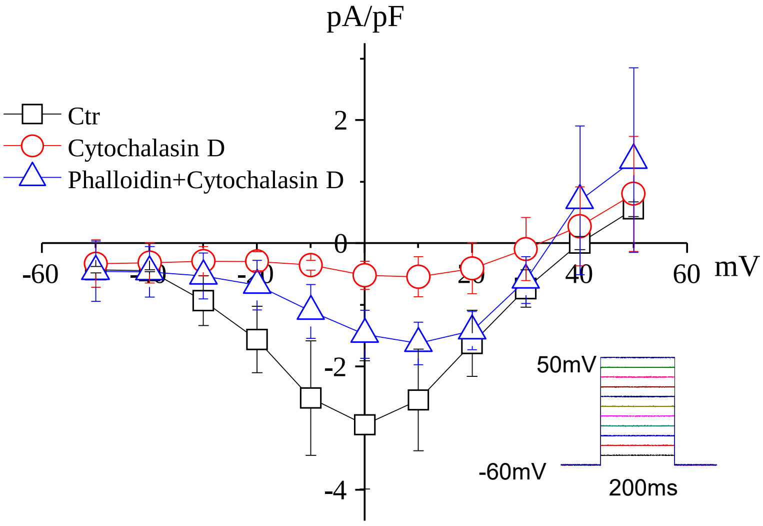

Supplementary Fig. 7. Effects of cytochalasin D and phalloidin on the voltage-dependent Ca^{2+} channels current recorded in A7R5 cells. The average I-V relationships were obtained by applying step pulses, the shape of which is shown as a figure inset, 10 min after whole cell formation. Cytochalasin D (10 μM) and phalloidin (10 μM) were added into the pipette solution (control, n = 4; cytochalasin D, n = 4; cytochalasin D + phalloidin, n = 4).

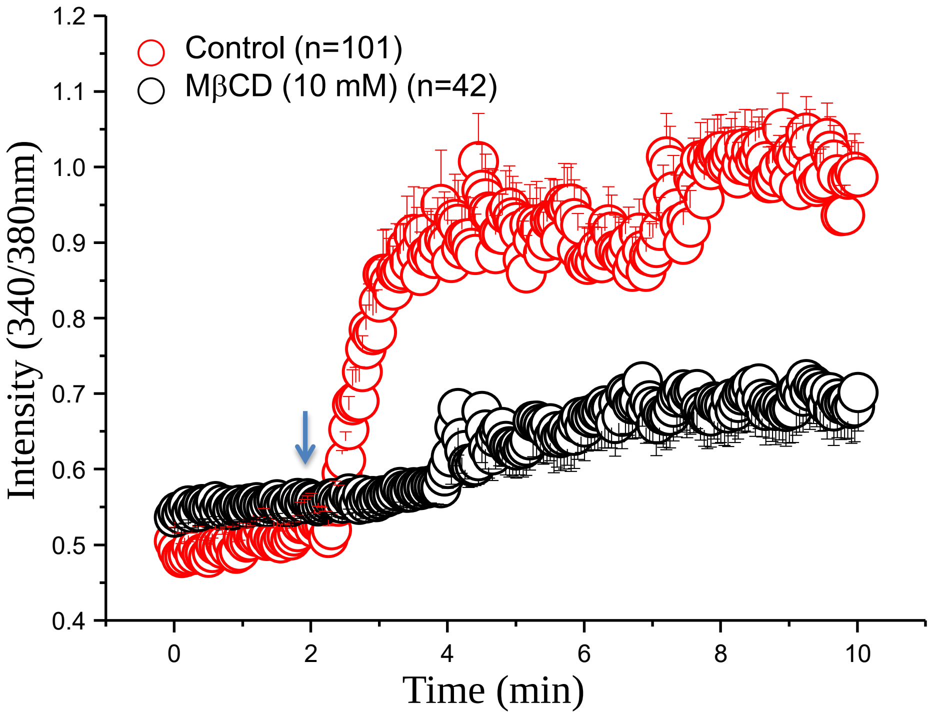

Supplementary Fig. 8. Effects of MβCD on the antigen-stimulated $[Ca^{2+}]_i$ increase in RBL2H3 cells. Summary of the antigen-stimulation effect on $[Ca^{2+}]_i$ increase in control and MβCD-pretreated cells. Stimulation with antigen (indicated with blue arrow) markedly increased $[Ca^{2+}]_i$ in control cells, whereas it had a minimal effect on $[Ca^{2+}]_i$ in MβCD-pretreated cells. n = 101 and n = 41 cells for the control and MβCD pretreatment groups, respectively.

References

1. Seebeck J, Tritschler S, Roloff T, Kruse ML, Schmidt WE, Ziegler A. The outwardly rectifying chloride channel in rat peritoneal mast cells is regulated by serine/threonine kinases and phosphatases. *Pflugers Arch.* 2002, **443**(4):558-564.
2. Kato N, Nakanishi M, Hirashima N. Cholesterol depletion inhibits store-operated calcium currents and exocytotic membrane fusion in RBL-2H3 cells. *Biochemistry.* 2003, **42**(40):11808-11814.
3. Rueckschloss U, Isenberg G. Cytochalasin D reduces Ca2+ currents via cofilin-activated depolymerization of F-actin in guinea-pig cardiomyocytes. *J Physiol*. 2001, **537**(Pt 2):363-370.
